# Differences in Dietary Preferences, Personality and Mental Health in Australian Adults with and without Food Addiction

**DOI:** 10.3390/nu9030285

**Published:** 2017-03-15

**Authors:** Tracy Burrows, Leanne Hides, Robyn Brown, Christopher V Dayas, Frances Kay-Lambkin

**Affiliations:** 1 Faculty of Health and Medicine, University of Newcastle, Callaghan NSW 2308, Australia; christopher.dayas@newcastle.edu.au (C.V.D.); f.kaylambkin@unsw.edu.au (F.K.-L.); 2 Centre for Youth Substance Abuse Reserach, School of Psychology, University of Queensland, St Lucia 4072, Australia; l.hides@uq.edu.au; 3 The Florey Institute of Neuroscience and Mental Health, Melbourne 3052, Australia; robyn.brown@florey.edu.au

**Keywords:** diet, food addiction, depression, obesity

## Abstract

Increased obesity rates, an evolving food supply and the overconsumption of energy dense foods has led to an increase in research exploring addictive eating behaviours. This study aimed to investigate food addiction in a sample of Australian adults using the revised Yale Food Addiction Survey (YFAS) 2.0 tool and how it is associated with dietary intake, personality traits and mental health issues. Australian adults were invited to complete an online survey that collected information including: demographics, dietary intake, depression, anxiety, stress and personality dimensions including impulsivity, sensation seeking, hopelessness and anxiety sensitivity. A total of 1344 individuals were recruited with the samples comprising 75.7% female, mean age 39.8 ± 13.1 years (range 18–91 years) and body mass index BMI 27.7 ± 9.5. Food addiction was identified in 22.2% of participants using the YFAS 2.0 tool, which classified the severity of food addiction as “mild” in 0.7% of cases, “moderate” in 2.6% and “severe” in 18.9% of cases. Predictors of severe food addiction were female gender (odds ratio (OR) 3.65 95% CI 1.86–7.11) and higher levels of soft drink OR 1.36 (1.07–1.72), confectionary consumption and anxiety sensitivity 1.16 (1.07–1.26). Overall people with “severe” (OR 13.2, 5.8–29.8) or extremely severe depressive symptoms (OR 15.6, range 7.1–34.3) had the highest odds of having severe food addiction. The only variable that reduced the odds of having severe food addiction was vegetable intake. The current study highlights that addictive food behaviours are associated with a complex pattern of poor dietary choices and a clustering with mental health issues, particularly depression.

## 1. Introduction

There has been a resurgence of interest in the potential addictive aspects of overeating [1]. This has largely been driven by increasing rates of obesity and the need to improve our current understanding of how to best treat obesity and address the obesogenic food environment (e.g., food marketing, advertising), prompting the over-consumption of readily available food [2,3]. Population consumption of energy-dense nutrient-poor foods exceeds national recommendations comprising approximately 40% of total daily energy intake [4]. In animal models, consumption of high-fat, high-sugar foods produced neurobiological and behavioural changes that closely parallel those observed after consumption of addictive drugs. While relatively few studies on food addiction in humans have been conducted to date, strong associations with reward dysfunction [5,6] and behavioral factors including cravings and impulsivity [7,8,9] have been found. Furthermore, food addiction in adults is associated with elevated body mass index (BMI) [10] and increased visceral adiposity [11]. However, the concept of food addiction remains a controversial topic, as there is no consensus definition of addictive-like eating in humans.

The Yale Food Addiction Scale (YFAS) first published in 2009, is a commonly used self-report measure of food addiction in adults and children [12,13]. While several other survey tools exist that assess addictive eating behaviours including the Food Craving Questionnaire [14], Food Craving Inventory [15] and Dutch Eating Behaviour Questionnaire [16], the YFAS remains the only measure of food addiction mapped directly from Diagnostic and Statistical Manual of Mental Disorders (DSM) criteria for substance use disorders [12]. The YFAS was recently updated to be consistent with DSM 5 criteria for substance use disorder and classifies the severity of food addiction as “mild”, “moderate” and “severe”. Systematic reviews of studies using the YFAS to assess food addiction indicate that this condition affects 20% of the population [10]. While there is a demonstrated significant overlap with obesity, binge eating and some mental health conditions, food addiction does not entirely explain these conditions.

Despite this work, the link between food addiction and dietary behaviour remains controversial. Research suggests that food addiction is associated with increased intakes of fat and protein [17]. A US study has indicated processed foods, higher in fat and glycaemic load [18], were associated with problematic, addictive-like eating behaviors, while another study in young adults 18–35 years found that it was associated with higher intakes of energy-dense nutrient-poor foods including confectionary and take away [19]. A case study of potential “cola dependence” demonstrated that YFAS scores reduced with a reduction in the amount of the cola consumed [20]. In addition, in a clinical population of obese individuals awaiting bariatric surgery, those classified as food addicted displayed greater pre-bariatric surgery cravings of starchy foods and fast foods [21]. Existing research is largely limited to young adults [19], undergraduate student samples [18] or clinical groups [20,21] and is yet to explore the association between food addiction measured using the YFAS 2.0 tool and comprehensive assessments of dietary intake.

Personality dimensions including anxiety sensitivity, negativity/hopelessness, sensation seeking and impulsivity have been associated with alcohol and drug misuse [22], but have received less attention in the context of food addiction. Specific personality dimensions have been associated with heightened motivations for substance use and comorbid mental health concerns. For example, people with high anxiety sensitivity are more likely to misuse alcohol to reduce anxiety [23]. Those scoring higher on hopelessness are more likely to experience major depressive episodes and to drink alcohol to relieve negative moods. Impulsivity has been associated with early onset and problematic substance use [24]. Finally, sensation seeking has the strongest links with alcohol (especially binge drinking) and drinking for positive reinforcement [24].

The relationship between personality and food use is complex and personality risk factors are likely to interact with substance use, mental health issues and stress to influence coping skills and subsequent risk of food addiction. Previous literature has shown that food addiction is associated with higher levels of impulsivity [7]; however, the link between other personality dimensions and food addiction is yet to be explored.

The aim of this study was to investigate food addiction in a sample of Australian adults using the revised YFAS 2.0 tool and how it is associated with dietary intake, personality traits and mental health issues.

## 2. Materials and Methods

### 2.1. Participants

Participants aged 18 years or above and living in Australia were invited to complete an anonymous online survey on food addiction. Pregnant and lactating women and people who could not comprehend English were ineligible for the study. Recruitment occurred from April to July 2016 (approx. three months). The study was advertised through media releases through three universities situated in both urban (Sydney and Melbourne) and regional areas (Newcastle). Emails were sent out through staff and student email lists in addition to advertisements via a variety of university social media platforms (i.e., Facebook, Twitter). “Virtual snowballing” was used across these mediums where participants could share or forward the study information. The advertisements contained a link to an information statement about the study that described the study purpose of researchers aiming to find out about food addiction in Australian adults. The advert also contained a link to the survey, and participants provided informed consent before completing the survey. If participants did not want to participate, they could opt out of the survey at any time. The survey took approximately 20 min to complete. Survey completers were invited to enter a prize drawing to win one of ten $50 shopping vouchers. This study was approved by the University of Newcastle Ethics Committee (H2016-010). The survey was pilot tested to check for readability, flow, spelling, grammar and ambiguity in questions with only minor changes required to achieve improved flow and readability.

### 2.2. Measures

Demographics: (10 items), which included information on gender, age, ethnicity, marital status, postcode, highest level of education, height and weight which converted into body mass index (BMI) using standardised equations. Postcode was used to determine the Index of Relative Socioeconomic Advantage and Disadvantage (ISRAD), where postcode is rated from one (most disadvantage/least advantage) to ten (least disadvantaged/most advantaged).

Food Addiction was assessed using the 35-item YFAS 2.0 [25]. This scale maps to DSM 5 criteria to assess for the 12 symptoms of substance use disorder, as well the level of distress associated with them. It provides a “diagnosis” of food addiction that can be classified as “mild”, “moderate” or “severe”. A “mild diagnosis” score is given when three or more symptoms are reported, moderate when 4–5 symptoms are present and severe when six or more symptoms are reported. Symptoms include tolerance, withdrawal, and loss of control with respect to eating behaviour. The YFAS 2.0 asks participants to think of specific foods such as highly processed foods; however, participants in this study were asked to consider all food. Food addiction questions were asked prior to the dietary intake questions to reduce bias.

Dietary intake (20 items) was assessed using standardised questions derived from the New South Wales Health Survey [26]. Participants were asked about their usual intake of: core foods including fruits and vegetables, which also included visual aids/pictures to represent a serve to improve estimation, breads, pasta, rice and cereals as well as milk intake. Food behaviours were also assessed including the regularity of breakfast intake and food insecurity, defined as “running out of food” or “couldn’t afford to purchase foods”. The weekly frequency (“less than once per week” to “six or more times per week”) of discretionary food consumption choices were also assessed, including fast food/take out, sweetened beverages (soft drinks and fruit juice), and snack foods (cakes, pastries, biscuits fries/hot chips, potato crisps, confectionary and ice cream).

Substance Use Risk Profile Scale (SURPS) [24]: The 23-item SURPS uses a four-point Likert scale (1 = strongly disagree to 4 = strongly agree) to assess four personality dimensions including: (1) anxiety sensitivity (a tendency to believe sensations of anxiety and fear will lead to illness embarrassment or heightened anxiety (e.g., “It’s frightening to feel dizzy or faint”); (2) hopelessness/depression proneness (a tendency to experience relatively frequent depressive symptoms) (e.g., “I feel that I am a failure”); (3) impulsivity (e.g., “I usually act without stopping to think”) and (4) sensation seeking (e.g., “I would like to skydive”).

Depression Anxiety and Stress Scale (DASS): The DASS 21 is a 21-item self-report measurement of the severity of depression (e.g.,” I felt down-hearted and blue”), anxiety (e.g., “I felt I was close to panic”) and stress (e.g.,” I found it hard to wind down”) in the past week. Each item is scored from 0 (did not apply to me at all over the last week) to 3 (applied to me very much or most of the time over the past week). The DASS is not a diagnostic tool and has demonstrated good psychometric properties with internal consistency estimates ranging from 0.82–0.93 [27]. Following author instructions, “mild” depression is classified with a scale score of 10–13, “moderate” 14–20, severe 21–27, “extremely severe” 28+. For anxiety: “mild” score is 8–10, “moderate” 12–14, “severe” 16–18, and “extremely severe” 20+. For stress: “mild” 16–18, “moderate” 20–24, “severe” 26–32, and “extremely severe” 34+.

### 2.3. Statistical Analysis

Participants with and without food addiction were compared by age group (18–34, 35–54 and 55+y), gender and dietary intake variables using ANOVA and chi-square statistics. Data is displayed as means ± SD. Correlations between continuous variables were determined using Pearson correlations, with the *p*-value set at < 0.05. A missing variables analysis was undertaken and it was identified that there was uniformity in the responses to the survey with 869 of the 1344 respondents who started the survey, answering all questions. Data was analysed using SPSS version 22.0 (SPSS Inc., Chicago, IL, USA).

Multinomial logistic regressions were then conducted to identify predictors of mild/moderate (*n* = 31) and severe (*n* = 168) food addiction relative to no food addiction (reference group). The low and moderate food addiction groups were combined due to the low number (*n*) of people with moderate food addiction. Initially, a correlation matrix was undertaken to identify which demographic, dietary intake, domain scores for mental health (depression, anxiety, stress) and personality variables were associated with the presence of mild/moderate or severe food addiction. Only variables with a correlation of >0.2 or highly significantly negative were included in the model. Several independent variables with high correlations (>0.6) were removed from the model to reduce risk of multicollinearity (e.g., *p* ≤ 0.001 for DASS “anxiety” and DASS “depression, *r* = 0.689, for snack foods and confectionary *r* = 0.495, *p* < 0.001). The remaining variables (gender, anxiety, vegetables, soft drinks, confectionaries and depression, were simultaneously entered into the regression model as either continuous or categorical depending on the lowest Akaike information criterion (AIC) value. Those which were not significant predictors of the presence of mild/moderate or severe food addiction relative to no food addiction were subsequently removed, and the models were re-run.

## 3. Results

### 3.1. Demographics

A total of 1344 individuals were recruited for the study, and demographic details of the sample are shown in Table 1. The sample was 75.7% female with the mean age of 39.8 ± 13.1 years (range 18–91 years). Only 2% identified as indigenous, and participants had a mean ISRAD score of 6.2 (range 1–10), indicative of moderate socioeconomic status. The mean BMI of the sample was 27.7 ± 9.5. Males (mean BMI = 28.4; SD = 6.2) reported higher BMIs than females (mean BMI = 27.5, SD = 10.1), but this difference was not statistically significant. Overall, 45.2% of the sample were in a healthy weight (BMI 18–24.9) range, 26% were overweight (BMI 25–30) and 28.8% were obese (BMI 30+).

### 3.2. Dietary Behaviour

A total of 47% of individuals reported consuming the recommended >2 servings of fruit per day; however, over half (53%) consumed <1 serving per day. Only 13.4% consumed the recommended >5 servings of vegetables per day. A total of 70% of people reported having breakfast everyday with 27% reporting consuming breakfast cereal daily, and 38.3% reported consuming breakfast cereal never or rarely. The two most popular milks consumed were regular full cream (38%) and low/reduced fat milk (29.9%). Take away food was consumed never or rarely by 76% of the sample, 19.2% consumed it 1–2 times per week and 1.3% reported 5–6 times per week. Most people (43%) reported consuming hot chips 1–2 times per week and over half (51%) consumed confectionary (chocolates/candy) three or more times per week. Most of the sample (82.6%) consumed less than one cup of soft drink per week, and 80% reported consuming less than one cup of fruit juice per week. Only 6.2% reported experiencing food insecurity in the past 12 months.

#### 3.2.1. Food Addiction

Across the whole sample food addiction was found to be 22.2% (*n* = 228). This differed significantly by gender, with females (24.4%) having a higher percentage than males (13.3%; *p* < 0.001). The rates of food addiction did not differ significantly by age group (*p* > 0.05). Of the 228 participants with food addiction, 0.7% (*n* = 7) were classified as mild, 2.6% (*n* = 27) moderate, and 18.9% (*n* = 194) severe. A similar pattern of distribution of the categories of food addiction was found to exist across all age groups and gender with the majority of those classified as food addicted classified as either mild or severe with very few in the moderate category. Cronbach’s alpha of the YFAS 2.0 in this sample was 0.95. The mean (±SD) number of food addiction symptoms in the whole sample was 4.0 ± 2.8 out of a possible 12, and was 7.7 ± 2.1 and 2.8 ± 1.8 among those with and without food addiction.

#### 3.2.2. Associations with Weight

A moderate correlation was found between the YFAS 2.0 total symptom score and BMI, *r* = 0.38, *p* < 0.001. This correlation was stronger in males, *r* = 0.54, *p* < 0.001, than females, *r* = 0.36, *p* < 0.001 and was strongest in the oldest age group (55+ years), *r* = 0.54, *p* < 0.001, followed by the youngest 18–35 years, *r* = 0.48, *p* < 0.001, and middle age group 35–55 years, *r* = 0.39, *p* < 0.001.

#### 3.2.3. Associations with Diet

**Figure 1:**
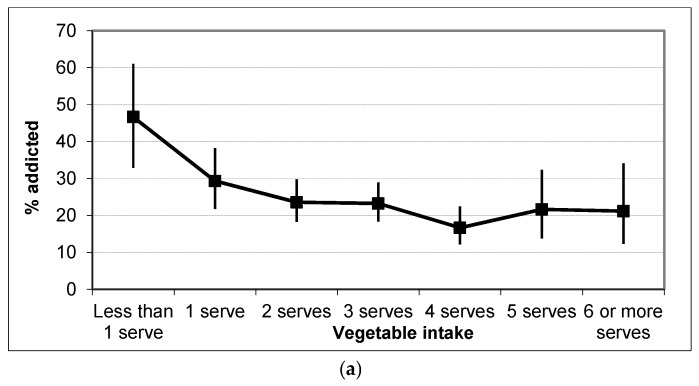

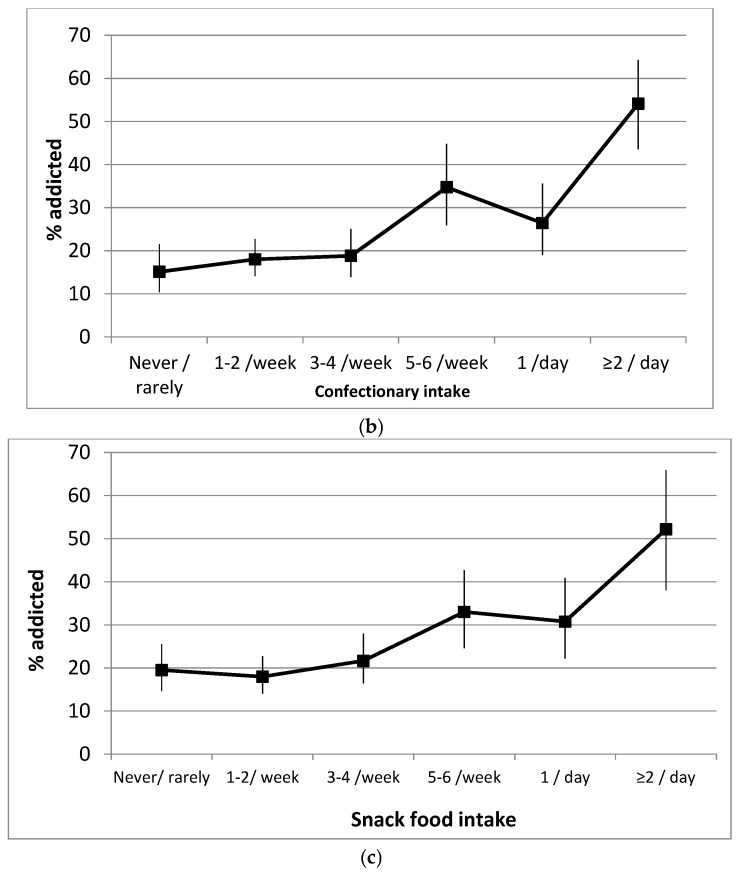
Percentage of food addicted individuals by frequency of core food group (**a**) vegetable intake; (**b**) confectionary intake; (**c**) snack food intake. Data is mean with bars representing the 95% CI.

The dietary intake of participants with and without food addiction was compared. A significantly higher proportion of people classified as food addicted reported higher intakes of confectionary, fast food, snack foods, hot chips, potato crisps and soft drinks, lower intakes of core foods like fruits, vegetables, and were less likely to report breakfast everyday than those without food addiction (Figure 1). No comparisons of the dietary intake of people with varying levels of food addiction were made, due to the low number of participants with moderate food addiction.

#### 3.2.4. Associations with Personality and Mental Health

Individuals with food addiction reported significantly higher scores on the depression, anxiety, and stress scales of the DASS 21 (Table 2). Cronbach alpha for the DASS and SURPS in this study were 0.94 and 0.67. People with food addiction had significantly higher anxiety sensitivity and impulsivity scores, and significantly lower hopelessness and sensation seeking scores. Of the individuals with food addiction, 84% (*n* = 169) were classified as having moderate /severe levels of stress, and 69% (*n* = 139) and 79% (*n* = 159) had high levels of anxiety and depression, respectively. The correlation between food addiction and stress was *r* = 0.449, food addiction with anxiety *r* = 0.488, and depression 0.514 were all statistically significant (*p* < 0.001). Twelve percent (*n* = 25) with food addiction had one concurrent mental health issue (classified as moderate or severe levels of depression, anxiety or stress), 23% (*n* = 47) had two concurrent mental health issues and 57% (*n* = 116) had three mental health issues, suggesting a clustering of conditions.

The results of the multinomial logistic regression are presented in Table 3. The final model included vegetables, soft drinks, confectionaries, DASS-depression and SURPS-anxiety sensitivity. DASS depression was the only variable that differentiated mild/moderate food addiction from no food addiction. Each one-point increase in the DASS depression score increased the odds of mild/moderate food addiction by a factor of 4.85. Significant predictors of severe food addiction included female gender, higher soft drink and confectionary intake, and higher levels of anxiety sensitivity. Females had 3.65 times the odds of severe food addiction, compared to males. Each one-point increase in anxiety sensitivity on the SURPS increased the odds of severe food addiction 1.6 times. Participants who ate confectionaries daily or 5–6 times per week had 2.4 times the odds of severe food addiction, while those who ate it two or more times a day had 7.1 times the odds of severe addiction. However, individuals with severe (OR = 13.2, range 5.8–29.8) or extremely severe depression (OR = 15.6, range 7.1–34.3) had the highest odds of severe food addiction, with each one unit increasing the odds by factors of 13.2 and 15.6, respectively. The only variable that reduced the likelihood of severe food addiction was vegetable intake, with each extra unit of vegetable consumption decreasing the odds by a factor of 0.8. This section may be divided by subheadings. It should provide a concise and precise description of the experimental results, their interpretation as well as the experimental conclusions that can be drawn.

## 4. Discussion

This study aimed to investigate food addiction in a sample of Australian adults, whether this varied by age group and gender, and how food addiction was associated with a range of mental health and personality variables. Overall, 22% of the 1344 survey respondents met criteria for food addiction on the YFAS 2.0. This rate is higher than the 14.6% rate of food addiction reported among 550 American adults in the original YFAS validation study [25], but is consistent with the 20% weighted mean percentage rate reported in a meta-analyses of 25 studies using the original YFAS tool [10]. Older adults (55+) had the highest rate of food addiction (24%), but no significant differences in the rates of food addiction were found between the three age groups examined (18–34, 35–54, 55+ years). Age was also not significantly associated with the presence of mild/moderate or severe food addiction in the regression analysis. Females (24.4%) were significantly more likely to have food addiction than males (13.3%; *p* < 0.001), and three times the risk of severe food addiction. These results are consistent with those reported in a meta-analysis of 25 studies [10].

When the YFAS 2.0 was used to classify food addiction as either mild, moderate or severe, the majority of individuals fell into a severe (18.9%) range with very few classified as mild (0.6%) or moderate (2.6%). Similar results were reported in the YFAS 2.0 validation study of 550 American with 1.7, 1.9 and 11.0% classified as mild, moderate and severe, respectively [25]. In the current study, similar proportions of mild, moderate and severe food addiction were found across the three adult age groups.

The mean number of food addiction symptoms reported across the entire sample was 4.0 ± 2.8 out of a possible 12. The only other study that used the YFAS 2.0 reported a lower mean symptom score of 2.4 [25]. This previous study had similar proportions of participants across the different weight categories (healthy weight, overweight, obese), with a similar BMI and also used an online survey to collect data. However, the current study had a higher proportion of female participants (75 vs. 54%), which is likely to account for this difference in results.

As expected, adults with a diagnosis of food addiction endorsed a significantly higher number of symptoms (mean = 7.7) on the YFAS 2.0. However, those who did not reach the threshold for food addiction reported a mean of 2.8 symptoms. This suggests that some food addiction symptoms including “increased attempts to cut down food” may not be specific to food addiction, and are characteristic of the eating habits of the majority of the general population who have attempted to reduce weight, particularly the 60% of adults who are overweight or obese [2]. Furthermore, the symptom of “loss of control with respect to eating behavior” overlaps with that of “loss of control over eating”, the latter being a core eating disorder behavior and a diagnostic criterion for the eating disorders bulimia nervosa and binge eating disorder [28].

Dietary variables were investigated as major food groupings of core foods such as fruits, vegetables breads, cereals, and breakfast consumption. Notably, people who reported a low consumption of health foods were more likely to be classified as food addicted versus those who consumed greater amounts. Similarly, consumption of less healthy foods including sweetened drinks, take out, and confectionary (chocolates and lollies) was associated with the presence of food addiction. Results from the regression analyses suggest that individuals with a high sugar intake, including confectionary and soft drink consumption, are at increased risk for severe food addiction, while those with a higher vegetable intake are at decreased risk of severe food addiction.

The current study highlights a complex pattern of poor dietary choices associated with addictive food behaviours. More individuals classified as food addicted reported a higher consumption of snack foods. While the categorisation of “snack foods” can be highly subjective, participants in this study were prompted with information about which foods are most likely to be consumed as snacks (e.g., sweet and savoury biscuits, cakes, donuts, or muesli bars). Taken together, a broader dietary pattern appears to be associated with food addiction. This may have relevance for treatments for food addiction. Individuals with food addiction were less likely to consume breakfast regularly, most often at consumption rates of 1–2 per week. Food addiction was not associated with food security, which was not surprising given the moderately high SES status of the group. Together, these results highlight the need for future research investigating the broader dietary patterns associated with food addiction.

Negative emotions are one of the most important triggers of self-regulation failure, that is, when people get upset, they comfort themselves with food, alcohol, or drugs. People with food addiction had significantly higher symptom levels of depression, anxiety and stress. Despite this, individuals with food addiction had only scores equivalent to moderate levels of depressive and anxiety symptoms, and mild levels of stress. Depressive symptoms were highest in those with mild and severe food addiction, while anxiety was highest in those with severe food addiction only. Combining these results with the dietary patterns associated with severe food addiction (e.g., low rates of regular breakfast consumption), these results may reflect an attempt to stimulate brain reward pathways to alleviate low food intake during times of depression. Anxiety sensitivity, a trait-like characteristic that increases an individuals’ propensity to experience anxiety, was associated with increased risk of mild, moderate and severe food addiction. In contrast, individuals with food addiction had significantly lower levels of trait hopelessness, which increases vulnerability to depression. Together, these findings suggest that anxiety symptoms may be an important target for interventions targeting food addiction. From the regression models, higher scores on the scale of the SURPS tool for anxiety sensitivity increased odds of food addiction, such that for every three-point increase, there was 59% more chance of classification of severe food addiction. The strongest predictor of severe food addiction was depressive symptoms, and the more severe the depression, the more likely an individual was to be classified as having a severe food addiction.

There is a considerable body of evidence indicating that the impulsivity and sensation-seeking people are associated with increased risk of alcohol and drug related problems [29]. Among people with food addiction, impulsivity may make it more difficult for people to resist the temptation to eat too much, while sensation seekers may pursue the sensory pleasure of eating food or novel and new food experiences. In the current study, people with food addiction were significantly more likely to be impulsive, but were less likely to be sensation seekers than those without food addiction. While both impulsivity and sensation seeking differentiated individuals with mild/moderate and severe food addiction from those without food addiction, the magnitude of difference was the smallest across all personality traits assessed. Dispositional impulsivity may thus be an important risk factor when considering tendency to engage in addictive consumption of food. The results of the study demonstrate the complexities around dietary behaviours and mental health.

Mental health also includes disordered eating, and previous research suggests that food addiction presents the most overlap with binge eating disorder. The overlap, however, is not 100% [30], and, while binge eating is a key eating disorder feature, the association between food addiction and eating-disordered behavior is unclear. It is acknowledged that the symptoms of general psychological distress found to be associated with food addiction in the present study are strongly associated with binge eating and other eating disorder symptoms [31].

Given that the current study is cross-sectional, causality cannot be inferred; however, it may be likely that an initial low depressive mood leads to a change in dietary intake (decreased intakes of core foods including vegetables and increases in non-core foods/drinks) contributing to an increase in anxiety, which, in turn, may contribute to subtle changes in impulsive eating contributing to overall increased symptoms of food addiction, which cycles back to low mood.

Limitations of the current study include its cross-sectional nature, the use of an online survey and its reliance on self-report data that is not verified by objective data (e.g., weight) or collateral reports. Therefore, some measures such as dietary intakes may be underreported due to social desirability. Both male and female individuals who completed the survey may have been motivated or interested in food and health, which may have contributed to selection and non-response bias that may impact findings. The majority of participants were female, which limits generalisability of results, and this sample is not considered representative, although it should be noted that the current study has more males than most other food addiction studies. The rates of food addiction observed in the current study sample should not be taken to infer the prevalence of food addiction in the population sampled. Future research should consider how mental health, personality, stress and coping interact to influence food addiction and include a more comprehensive measure of impulsivity and sensation seeking. In addition, comprehensive assessment of eating disorder symptoms should be investigated in future studies of food addiction and be considered alongside developments in the eating disorders literature including efforts to improve public awareness and understanding [32].

## 5. Conclusions

This study found that 22.2% of an Australian based sample of adults met criteria for food addiction, the majority of whom were classified with “severe” food addiction. Depression was the strongest predictor of severe food addiction, which was also associated with anxiety sensitivity, female gender, confectionary and soft drink intake. Vegetable intake decreased the risk of severe food addiction. While prospective research is needed, these results highlight a number of variables that may contribute to the risk of food addition, among a sample Australian adults, and be important targets for treatment.

## Figures and Tables

**Table 1:** Demographics of participants by age group.

Characteristic	18–34 Years			35–54 Years			55+ Years		
	Male	Female	Total	Male	Female	Total	Male	Female	Total
	*n*	*n*		*n*	*n*		*n*	*n*	
**Gender**	88 (16%)	462 (84%)	*n* = 550	107 (22%)	382 (78%)	*n* = 489	43 (24%)	139 (76%)	*n* = 182
**BMI (kg/m^2^)**	26.7 ± 5.57	25.2 ± 6.49	25.4 ± 6.4	29.2 ± 5.82	29.5 ± 13.6	24.5 ± 12.4	30.08 ± 7.7	29.4 ± 7.1	29.6 ± 7.2
**Healthy weight (%)**	44.8	63.5	60.4	21.9	38.0	34.4	26.8	30.6	27.5
**Overweight (%)**	34.5	19.2	21.6	41.9	26.0	29.5	29.3	30.6	30.3
**Obese (%)**	20.7	17.4	17.9	36.2	36.1	36.1	43.9	41.8	42.3
**SES (ISRAD) ^1^**		6.2 ± 2.9			6.2 ± 2.8			5.9 ± 2.6	
**Food addiction ^2^ (% Addicted)**	12.2	23.7	21.4	12.6	24.9	22.3	16.7	26.6	24.1
- **Mild**	0	0.5	0.4	0	1.3	1.0	0	0.8	0.6
- **Moderate**	2.7	2.9	2.9	2.3	3.8	3.5	0	0.0	0
- **Severe**	9.5	19.7	18.1	10.3	19.9	17.8	16.7	25.8	23.5
**Total FA symptoms (Range 0–12)**	3.5 ± 2.39	4.06 ± 2.93	4.0 ± 2.9	3.21 ± 2.36	4.14 ± 2.83	3.9 ± 2.8	3.64 ± 2.5	4.36 ± 2.86	4.2 ± 2.8

^1^ SES assessed by ISRAD scale (1–10 with 1 being most disadvantages–10 being least disadvantaged); ^2^ Food addiction assessed by the YFAS 2.0.

**Table 2:** Personality traits of Australian adults by food addiction categorisation determined by YFAS.

Variables	Not Food Addicted *n* = 659	Food Addicted *n* = 202	*p* Value	Mild *n* = 7	Moderate *n* = 24	Severe *n* = 171	*p*-Value
**Food Addiction Symptoms**	2.83 ± 1.8	7.72 ± 2.11	<0.001	2.71 ± 0.49	4.56 ± 0.51	8.35 ± 1.57	<0.001
**DASS depression (total domain score)**	6.9 ± 6.8	15.7 ± 10.4	<0.001	17.4 ± 10.1	10.2 ± 8.1	16.5 ± 10.5	<0.001
- **Mild (%)**	10.5	9.4	<0.001	14.3	20.8	7.6	<0.001
- **Moderate (%)**	9.9	27.7		28.6	25	28.1	
- **Severe (%)**	2.0	12.2		14.0	0	14	
- **Extremely severe (%)**	2.1	16.8		14.3	4.2	18.7	
**DASS anxiety (total domain score)**	4.3 ± 4.5	9.8 ± 7.4	<0.001	7.7 ± 6.5	5.6 ± 4.9	10.8 ± 7.5	<0.001
- **Mild (%)**	12.1	14.9	<0.001	14.3	29.2	12.9	<0.001
- **Moderate (%)**	5.9	15.8		28.6	4.2	17	
- **Severe (%)**	1.5	10.9		14.3	0	12.8	
- **Extremely severe (%)**	1.4	12.9		0	4.2	14.6	
**DASS Stress (total domain score)**	9.7 ± 7.1	17.3 ± 8.8	<0.001	15.4 ± 9.6	13.6 ± 8.1	17.9 ± 8.8	<0.001
- **Mild (%)**	9.7	14.4	<0.001	14.3	20.8	13.5	<0.001
- **Moderate (%)**	7.9	17.8		0	12.5	19.3	
- **Severe (%)**	2.7	19.8		28.6	4.2	21.6	
- **Extremely severe (%)**	0.3	3.0		0	4.2	2.9	
**SURPS**							
**Hopelessness**	18.62 ± 3.1	15.37 ± 3.7	<0.001	14.29 ± 4.27	17.25 ± 3.4	15.14 ± 3.66	<0.001
**Anxiety sensitivity**	11.86 ± 2.69	13.37 ± 2.86	<0.001	11.86 ± 3.02	12.67 ± 3.13	13.53 ± 2.79	<0.001
**Impulsivity**	9.85 ± 2.42	10.95 ± 2.65	<0.001	11 ± 4.32	10.79 ± 2.64	10.98 ± 2.58	<0.001
**Sensation Seeking**	12.21 ± 3.29	10.91 ± 3.32	<0.001	11.86 ± 1.95	12.96 ± 3.41	10.58 ± 3.25	<0.001

Data is displayed as mean ± SD.

**Table 3:** Predictors of severe food addiction (*n* = 168).

Variable	B (S.E)	OR (95% CI)	*p*-Value
**Anxiety sensitivity ^a^**	0.155 (0.041)	1.59 ( 1.25–2.03) *	**<0.001**
**Vegetable consumption**	−0.205 (0.068)	0.81 (0.71–0.93)	**0.003**
**Soft drink consumption**	0.307 (0.121)	1.36 (1.07–1.32)	**0.011**
**Females**	−1.294 (0.341)	3.65 (1.86–7.11)	**0.000**
**Depression Nil ^b^**		-	
**Depression Mild**	0.440 (0.356)	1.55 (0.77–3.12)	0.217
**Depression Moderate**	1.885 (0.263)	6.58 (3.93–11.04)	**0.000**
**Depression Severe**	2.581 (0.416)	13.2 (5.84–29.83)	**0.000**
**Depression Extremely severe**	2.747 (0.402)	15.59 (7.1–34.3)	**0.000**
**Confectionaries Never or rarely**	-	-	-
**Confectionaries 1–2 per week**	0.402 (0.371)	1.49 (0.72–3.09)	0.279
**Confectionaries 3–4 per week**	0.170 (0.404)	1.18 (0.54–2.61)	0.673
**Confectionaries 5–6 per week**	0.914(0.432)	2.4 (1.1–5.8)	**0.034**
**Confectionaries 1 per day**	0.891(0.419)	2.43 (1.1–5.5)	**0.033**
**Confectionaries ≥2 per day**	1.963(0.417)	7.1 (3.1–16.1)	**0.000**

Reference group: no food addiction (*n* = 656). Bold text represents statistically significant *p* < 0.05. * This odds ratio equates to every three-point increase in anxiety sensitivity score, and all others are equivalent to a one-point increase. ^a^ Anxiety sensitivity was assessed by the SURPS tool, ^b^ Depressive symptoms as assessed by the DASS tool Tables may have a footer.
